# Boerhaave syndrome and black esophagus: Síndrome de Boerhaave e esôfago preto

**DOI:** 10.1590/1516-3180.2016.0354020117

**Published:** 2017-04-20

**Authors:** Grigoriy Emil Gurvits

**Affiliations:** I MD, FACP, FACG. Clinical Associate Professor, Department of Medicine, Division of Gastroenterology, New York University School of Medicine/Langone Medical Center, New York, United States.

Dear Editor,

It was with great interest that I read an article by Dr. Dinic and colleagues on Boerhaave syndrome in the latest issue of your journal.^1^ They describe a rare case of spontaneous esophageal perforation in the setting of hematemesis in association with duodenal ulcer and black esophagus.

Boerhaave syndrome is an unusual entity in clinical medicine that was historically described by classical physical examination findings of the Mackler triad (vomiting, chest pain and subcutaneous emphysema), Hamman’s mediastinal crepitus with heartbeat and pneumomediastinum on X-ray imaging. Computed tomography scans showing Gastrografin extravasation are diagnostic on call to the operating room.^2^ Patients typically appear tachypneic and acutely ill.

The case of esophageal perforation presented by Dinic et al. is intriguing for several reasons, primarily because of the presence of a rare finding of distal black esophagus on postmortem examination. Black esophagus or acute esophageal necrosis is a syndrome that is classically characterized by a distal esophagus with circumferential black appearance of varying proximal extent. It presents clinically with gastrointestinal hemorrhage and is often associated with duodenal ulcer disease.^3^^,^^4^^,^^5^ The patient’s clinical history and a four day prodrome may be a clue to the development of black esophagus.

It is true that profound retching with a sudden rise in intraesophageal pressure against a closed cricopharyngeus may result in spontaneous distal esophageal perforation, as seen in Boerhaave syndrome. On the other hand, blind passage of the nasogastric tube in the setting of a necrotic esophagus would potentially cause a longitudinal tear of similar appearance at the weak point just above the gastroesophageal junction. In fact, such a procedure should be avoided.^4^ It is also conceivable that repeated vomiting in a patient with black esophagus may result in Boerhaave syndrome. If so, this would be the first such occurrence described in the literature.

It is difficult to retrospectively point out the correct underlying diagnosis in this unfortunate case, but the possibility of an iatrogenic esophageal tear should be considered in this patient with concurrent finding of black esophagus.
