# Supplementary material for: Boerhaave syndrome and black esophagus: Síndrome de Boerhaave e esôfago preto
Source: Sao Paulo Med J. 2017 Apr 20;135(2):190–1. doi: 10.1590/1516-3180.2016.0354020117 (PMC9977335; doi:10.1590/1516-3180.2016.0354020117)
Supplement: Related texts

# Reply: RESPONSE TO LETTER TO THE EDITOR

**Authors:** Biljana Radovanovic Dinic, Goran Ilic, Snezana Tesic Rajkovic, Tatjana Jevtovic Stoimenov

**Affiliations:** I MD. Associate Professor and Attending Physician, Medical School, University of Niš, and Gastroenterology and Hepatology Clinic, Niš Clinical Center, Niš, Serbia.; II MD. Associate Professor, Medical School, University of Niš, and Institute of Forensic Medicine, Niš, Serbia.; III MD. Attending Physician, Gastroenterology and Hepatology Clinic, Niš Clinical Center, Niš, Serbia.; IV MD. Associate Professor, Medical School, University of Niš, and Institute of Biochemistry, Niš, Serbia.

Dear Sir,

I am glad that our work has sparked your interest. The study^1^ presents a case of a patient with spontaneous rupture of the esophagus caused by vomiting. In Figure 2, we presented the esophagus with its surface covered with blood (not washed). The wall of the esophagus was not necrotized (shown histopathologically), and it was not a black esophagus.^2^^,^^3^ Setting the nasogastric tube in this case was risky; however, it was necessary in order to evacuate the heavy liquid content of the stomach (detected on abdominal ultrasound), as well as to perform esophagogastroduodenoscopy (EGD). The patient had the same symptoms before placing the probe and after removing it (before EGD), and so we ruled out the possibility that she might have caused the rupture herself.

## References

[1] Dinic BR, Ilic G, Rajkovic ST, Stoimenov TJ (2016). Boerhaave syndrome - case report. Sao Paulo Med J.

[2] Kimura Y, Seno H, Yamashita Y. (2014). A case of acute necrotizing esophagitis. Gastrointest Endosc.

[3] Gurvits GE, Cherian K, Shami MN (2015). Black esophagus: new insights and multicenter international experience in 2014. Dig Dis Sci.
